# FACEPAI: a script for fast and consistent environmental DNA processing and identification

**DOI:** 10.1186/s12898-019-0269-1

**Published:** 2019-12-06

**Authors:** Emma Wahlberg

**Affiliations:** 1 Department of Zoology, Stockholm University, Svante Arrhenius väg 18b, 106 91 Stockholm, Sweden; 2 Department of Zoology, Swedish Museum of Natural History, P. O. Box 50007, 104 05 Stockholm, Sweden

**Keywords:** eDNA, Filtering sequence reads, BLAST, Identification, Bash script, Bioinformatics

## Abstract

**Background:**

The use of environmental DNA (eDNA) has become an increasing important tool in environmental surveys and taxonomic research. High throughput sequencing of samples from soil, water, sediment, trap alcohol or bulk samples generate large amount of barcode sequences that can be assigned to a known taxon with a reference sequence. This process can however be bioinformatic cumbersome and time consuming, especially for researchers without specialised bioinformatic training. A number of different software packages and pipelines are available, but require training in preparation of data, running of analysis and formatting results. Comparison of results produced by different packages are often difficult.

**Results:**

FACEPIE is an open source script dependant on a few open source applications that provides a pipeline for rapid analysis and taxonomic assignment of environmental DNA samples. It requires an initial formatting of a reference database, using the script CaPReSe, and a configuration file and can thereafter be run to process any number of samples in succession using the same settings and references. Both configuration and executing are designed to demand as little hands on work as possible, while assuring repeatable results.

**Conclusion:**

The demonstration using example data from real environmental samples provides results in a time span ranging from less than 3 min to just above 15 min depending on the numbers of sequences to process. The memory usage is below 2 GB on a desktop PC. FACEPAI and CaPReSe provides a pipeline for analysing a large number of eDNA samples on common equipment, with little bioinformatic skills necessary, for subsequent ecological and taxonomical studies.

## Background

The development in high throughput sequencing methods have contributed to an increasing interest in sequencing DNA not only from organismal tissue but also molecular fragments deposited in water, soil, sediment, faecal matter and stomach contents. Sequencing of fragments from trap alcohol and bulk samples of e.g. insects are also current research areas. Identification of these sequences may be carried out through taxonomic assignment by the aid of barcode sequence regions, a practice commonly known as metabarcoding [1]. The number of reads from these samples can reach millions, and clustering and assigning clusters to taxonomical units can become a daunting task demanding skilled bioinformatic expertise. There are a number of metabarcoding pipelines published, e.g. obitools [2], Bista et al. [3] and Hawlitschek et al. [4]. But very few published method offers a solution with a complete process including quality control, aligning, clustering and taxonomic assignment in an automatic process from raw input to results output (e.g. SLIM [5]). The purpose of the scripts described in this paper is to provide a reproducible and fast method for processing of raw metabarcoding data and assigning molecular Operational Taxonomic Units (MOTUs) to taxonomic reference data using established methods and applications in a easy-to-use and automated environment. Conversion and Preparation of Reference Sequences (CaPReSe) is a script preparing a reference sequence dataset from Barcode of Life Database (BOLD) or other source, for subsequent and repeatedly us in the script Fast And Consistent Environmental DNA Processing And Identification (FACEPAI). FACEPAI takes raw reads as input and process them and provides a table with taxonomic assignments. By providing these scripts metabarcoding methods can be utilized and data be processed in any setting, in private or governmental organisations, and without the requirement of bioinformatic expertise for carrying out routine processing of samples. The scripts have been developed and tested on a Linus system (Ubuntu 18.04.2 LTS), and other Unix systems have not been tested.

## Implementation

**Fig. 1:**
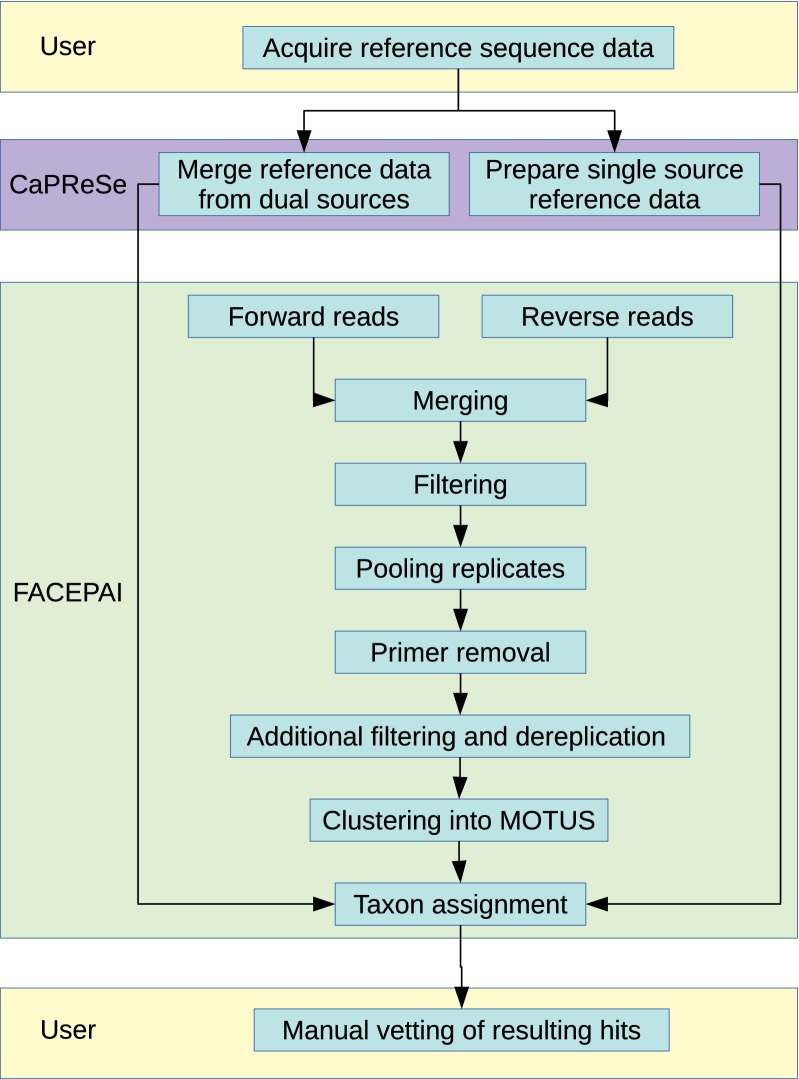
Flow chart representing the pipeline, and what steps are performed within CaPReSE, FACEPAI and by user

The scripts FACEPAI and CaPReSe are executed in Bash in a Linux environment. Examples of commands for running the script are included with the scripts. The most time-consuming part of the process is downloading the reference database. However, once that is accomplished, the process of first preparing the database using CaPReSe and thereafter using FACEPAI for pooling, merging, filtering and identifying eDNA sequences requires a minimum of hands on involvement (Fig. 1).

### Preparing reference database

The script FACEPAI is constructed to format a results table using a database file retrieved from BOLD. The standard FASTA file downloaded from BOLD will not include information about location and taxonomic lineage. Therefor it is recommended to download a TSV file (option “Combined: TSV” at BOLD website), and thereafter convert the TSV file to a FASTA file. The accessory script CaPReSe can be used to convert the TSV file to a FASTA file. The script will at the same time automatically filter out sequences that are not assigned to a Barcode Index Number (BIN) Uniform Resource Identifier (URI—a unique string identifying a resource), to assure that only validated quality sequences are kept. The BIN, in this case a BIN in the BOLD database, is a record associated with validated barcode sequences and represents a species. The argument ‘-C’ followed by the name of the source and filename will convert the file and add the name of the source as a source identifier for each reference sequence.

CaPReSe can also be used to merge and prepare two FASTA files with reference sequences for use with FACEPAI, keeping only unique sequences. CaPReSe will add identifiers (name of source above) making it easy to distinguish the sources in the results. This is useful for e.g. merging BOLD and private or GenBank data. A FASTA file converted from a TSV file in step 3 is an ideal source for BOLD data. A GenBank or private FASTA file needs to be prepared before acceptance. Header elements (designated by a ‘>’ according to FASTA format standard) must be delimited by a pipe sign (|), with GenBank or other ID first, followed by taxon name, and thereafter extra information (e.g. taxonomic linage). For creating a GenBank FASTA file with optional and customizable information it is recommended to download a GenBank (GB) file and use the script GenBank_to_FASTA created by McKay and Rocap [6]. The separator in the GenBank FASTA file should be a pipe sign, as in the BOLD FASTA file. CaPReSe is then executed with the argument ‘-M’ followed by name of first source, name of second source, first file name and second file name. It is highly recommended to construct a BLAST database from the reference FASTA file, this will drastically improve performance and memory use. A reference database should be restricted to the geographical region of interest and narrowed to relevant organism groups. This will further not only speed up the process but also return more precise matches. Recommended command for making a BLAST database, using makeblastdb, is ‘makeblastdb-in reference_fasta_file.fasta-title “name of database”-dbtype nucl’ [7].

### Configurating and running FACEPAI

Configuration is carried out by editing the variables in the file ‘options.config’. The variables that needs to be changed accordingly are forward primer, reverse primer, minimum length and the path to the reference reference sequences database. The reverse primer is entered reverse complement of the reverse primer. The table headers are left as default when following the above procedure to prepare the database. Detailed parameters and variables can be changed in the script, such as filtering procedure. FACEPAI is then executed in the Bash terminal from the folder containing the FASTQ files with sequencing reads from environmental samples. These reads must be demultiplexed in advance, either by the sequencing provider or by the user before running FACEPAI. The reads may come from one sample or may be consist of multiple files from several replicates of the same sample. In the second case these replicates will be pooled. FACEPAI is run with the sample name as first argument, followed by the ending of forward and reverse reads file names (e.g. ‘SampleName _F.fastq _R.fastq’).

The script FACEPAI first filters the forward and reversed reads for each replicate while at the same time merging them utilizing fastp [8] with paired ended base correction option to filter out low quality reads (standard setting is keeping reads with phred ≥ Q15, overlap difference limit 40%, overlap length require 10%). The individual replicates (if more than one replicate is available) are then pooled. The replicates, if multiple, are then pooled. Primers and reads below minimum length are removed using cutadapt [9], and the sequences are then filtered again using vsearch [10] to remove sequences with N:s. Sequences are then dereplicated before MOTUs are clustered using swarm [11], and checking for chimeras is carried out using vsearch. The resulting MOTUs are thereafter BLAST-searched against the database file using blastn [7]. The resulting hit table is then written to a tab delimited file with headers. Example files, both raw reads and resulting output files, are available in the GitHub repository together with detailed instructions for running the scripts in different situations.

## Results and discussion

Log files along with output files from e.g. fastp and cutadapt are written to the same folder as the raw read data. The tab (file suffix.tab) delimited hit table can be manually curated and checked for discrepancies and ambiguities in preferable spread sheet editor. FACEPAI return the top 10 hits for each sequence, to aid in the evaluation of each identification. If the recommended process for reference sequences file preparation described above is followed, the results will report a unique query sequence identifier, number of sequences included in the MOTU, identity in percent, e-value, query coverage in percent, source of subject (e.g. BOLD or GenBank if using concatenated files produced in CaPReSe), subject ID, BOLD BIN URI, taxon name, GenBank ID for BOLD subjects with corresponding GenBank data, country and taxonomic lineage. This may differ if another source or preparation of reference sequences are used, and if the heading settings are changed in the configuration file. The user may filter the table in preferred spreadsheet software depending on the research subject and hypothesis, e.g. by filtering on abundance units (number of sequences included in the MOTU), identity scores and location data. Some species may be very closely related, and even difficult to separate through molecular methods. Care should also be taken regarding errors in the reference database and with MOTUS with a low number of reads. Decisions like this during post-processing require taxonomical and biological expertise, especially if the reference database is broad and not geographically restricted. For statistical purposes additional files are produced by swarm, e.g. a file with the prefix.statsta. This file contains detailed numbers from the swarm process, and how to interprete is described in the swarm manual [11].

For demonstration and evaluation of time and memory consumption, three sets of environmental data were processed using FACEPAI. The environmental samples were taken in situ from sediment (‘Sed’), soil (‘Soi’) and water (‘Wat’) at a freshwater stream. Water samples (0.5 l per replicate) were filtered using Sterivex-HV Pressure 45 μm filter and thereafter lysed overnight using 600 μl lysis buffer and 75 μl Proteinase K from QIAGEN. KingFisher™ Duo (Thermo Scientific) extraction robot was used together with KingFisher™ Cell and Tissue DNA Kit (Thermo Scientific) for extraction after lysis. Soil and sediment samples (ca 0.25 g per replicate) were extracted using MagAttract PowerSoil DNA Kit (QIAGEN) together with the same extraction robot according to manufacturers protocol. The primers in this study were developed by Elbrecht and Leese [12]. The primer pair BF1 and BR2 targets a 316 bp long fragment of the second half of the 658 bp COI barcoding region. Library construction was performed using a standard barcoding amplicon PCR protocol and subsequent pooling before sequencing on a MiSeq Illumina system (v3). Both library preparation and sequencing were carried out at an external laboratory (Macrogen, Seoul, Korea). Each set consisted of three sample replicates of varying number of raw reads quantity (Table 1) (available as example data in the FACEPAI GitHub repository).

**Table 1:** Environmental DNA samples used for evaluation

Sample name	Total bases	Read count
Sed_1	61,880,182	205,582
Sed_2	22,210,188	73,788
Sed_3	42,460,866	141,066
Total	126,551,236	420,436
Wat_1	120,107,428	399,028
Wat_2	189,115,290	628,290
Wat_3	76,450,388	253,988
Total	385,673,106	1,281,306
Soi_1	546,386,638	1,815,238
Soi_2	580,222,048	1,927,648
Soi_3	180,489,232	599,632
Total	1,307,097,918	4,342,518

The processing was performed on a desktop computer with Intel^®^ Core™ i7-8700 CPU @ 3.20 GHz × 12 CPU, 64-bit architecture and with 16 GB RAM. Operative system was Ubuntu 18.04.2 LTS. Processing time for the samples varied from 2 min and 26 s to 15 min and 37 s, the memory usage reached 1519 MB at maximum (Table 2). The discrepancies in time was in large parts due to the time required for BLAST identification with increased number of MOTUs.

**Table 2:** Time and memory allocation for each sample processed, with all replicates pooled

Sample name	MOTU count	Time	Memory usage (MB)
Sed pooled	1150	2 m 26 s	874
Wat pooled	2144	7 m 7 s	1252
Soi pooled	8952	15 m 37 s	1519

The tab delimitated results table can be sorted and filtered using a spread sheet editor, and the results manually inspected by taxonomic or ecological expertise without special bioinformatic training. If combined reference sequences from different sources have been used detection of ambiguous reference data is more easily discovered. In contrast to existing scripts and pipelines, FACEPAI requires little knowledge in programming, statistical computing and only superficial command line skills. Furthermore, it utilizes applications with available source codes. Both the script and applications can be modified if needed, and applications may be exchanged as well with modifications of the script. Because of the low number of operations and commands required from the users for each individual run and the speed of the complete process, the repeatability of the analysis is high and human error is minimized. For projects with large number of samples, both repeatability and speed are of essence. The intended users are ecological, taxonomical and environmental scientists performing analysis of the taxonomic composition sourced from environmental DNA. Future plans of developments include updates of taxonomic assignment routines following current research, additional pre-filterings steps (e.g. abundance limits) and a graphical user interface to make the pipeline even more accessible.

## Conclusions

The script FACEPAI allow rapid and repeatable taxonomic assignment of molecular sequences acquired from environmental DNA, with its major strength in ease of use and automated process. This is important in the growing use of eDNA sequences and metabarcoding data from high throughput sequencing, with increasing size of databases as well as more complex bioinformatic routines. FACEPAI provides a rapid analysis for use in situations where time may be of importance, such as governmental or corporate environmental monitoring, and results interpretable by non-bioinformatics such as taxonomists or ecologists.

## Data Availability

The demonstration datasets generated and analysed during the current study as well as a comparison of a subset of data from a unrelated study [13] are available in the FACEPAI GitHub repository, https://github.com/emmawahl/facepai. Project name: FACEPAI; Project home page: https://github.com/emmawahl/facepai; Operating system(s): Linux; Programming language: Bash shell script; Other requirements: fastp 0.20.0 (should be installed with link in bin to allow global access), cutadapt 1.15, vsearch 2.7.1, swarm 2.2.2, blastn 2.6.0, makdeblastdb 2.6.0 (the scripts are tested with version numbers mentioned, they may work with both earlier and later versions); License: GNU General Public License version 3; Any restrictions to use by non-academics: no restrictions.

## Abbreviations

BIN: Barcode Index Number—a cluster of similar barcode references in the BOLD database, in high concordance with a species
BLAST: Basic Local Alignment Search Tool—a tool for comparing similar sequences based on alignment
BOLD: The Barcode of Life Database—a reference database with DNA sequence data
bp: basepair—the number of basepairs in a given sequence
eDNA: environmental DNA—fragments of DNA sequences deposited in the environment by living organisms
FASTA: the fast A file format for sequence data
FASTQ: the fast Q file format sequence data along with quality scores
MOTU: Molecular Operational Taxonomic Unit—a group of closely related individuals based on molecular data
TSV: tab-separated values—a text file of a dataset in a tabulated structure
URI: Uniform Resource Identifier—a unique string identifying a resource, in this case a BIN in the BOLD database
